# Hepatoprotective Effect of *Actinidia deliciosa* against Streptozotocin-Induced Oxidative Stress, Apoptosis, and Inflammations in Rats

**DOI:** 10.1155/2022/1499510

**Published:** 2022-03-19

**Authors:** Fatma M. El-Demerdash, Yousra Talaat, Raghda A. El-Sayed, Wenyi Kang, Nora F. Ghanem

**Affiliations:** ^1^Department of Environmental Studies, Institute of Graduate Studies and Research, Alexandria University, Alexandria, Egypt; ^2^National R & D Center for Edible Fungus Processing Technology, Henan University, Kaifeng 475004, China; ^3^Department of Zoology, Faculty of Science, Kafrelsheikh University, Kafr ElSheikh, Egypt

## Abstract

The present research intended to assess the possible protective and hypoglycemic effect of *Actinidia deliciosa* fruit aqueous extract (ADAE) in diabetic rats. The scavenging antioxidant capabilities of ADAE were evaluated using GC-MS analysis. In addition, rats were divided into four groups: control, ADAE, streptozotocin-induced DM (STZ), and STZ-treated rats + ADAE in an in vivo investigation. GC-MS analysis of ADAE was shown to include major components with high total phenolic contents and high DPPH scavenging activity. In diabetic rats, significant elevation in blood glucose level, lipid peroxidation, bilirubin, and lactate dehydrogenase activity as well as a change in lipid profile was observed, while insulin, body and liver weights, enzymatic and nonenzymatic antioxidants, liver function biomarkers, and protein content were significantly decreased. Furthermore, changes in the expression of the peroxisome proliferator-activated receptor (PPAR-*γ*), apoptotic, and inflammation-related genes were found. In addition, histological differences in rat liver tissue architecture were discovered, corroborating the biochemical modifications. However, consuming ADAE alone reduced lipid peroxidation and improved antioxidant status. Furthermore, diabetic rats given ADAE showed significant reductions in oxidative stress indicators and biochemical parameters, as well as improved tissue structure, when compared to the diabetic rats' group. Also, ADAE supplementation protects diabetic rats' hepatic tissue by upregulating PPAR-*γ* and downregulating apoptotic and inflammatory-related gene expression. In conclusion, *A. deliciosa* has beneficial protective effects so, it might be used as a complementary therapy in diabetes mellitus.

## 1. Introduction

Diabetes mellitus (DM) is one of the most prevalent diseases and one of the principal causes of mortality and morbidity in the world [1]. It is characterized by hyperglycemia because of the deficient action of insulin on the target organ or its secretion from pancreatic *β*-cell leading to metabolic disorder in proteins, carbohydrates, and fats; irreversible injury; and organ dysfunction [2]. The incidence of DM might be affected by several factors including aging, genetic and environmental factors, and lifestyle [3]. Several studies reported that the elevation of oxidative stress due to excess formation of reactive oxygen species (ROS) and the alterations of the antioxidant status are essentially related to diabetes complications [4]. Glucose-autooxidation and nonenzymatic protein glycosylation are the main sources of this free radical generation which leads to tissue damage in diabetic complications [5]. The major disorder of diabetes is well-known as hyperglycemia, hyperlipidemia, cardiovascular complications, nephropathy, and retina damage as well as liver dysfunction due to chronic hyperglycemia and increased oxidative stress [6].

There has been a growing interest in substituting manufactured antidiabetic medicines with plant antioxidants that can prevent the generation of advanced glycated end products and other diabetic complications associated with oxidative stress [7]. Medicinal plants have many properties such as effectiveness, safety, and low cost for many diseases. Owing to the presence of active constituents in medicinal herbs, they have been reported to acquire some characteristic properties like pancreatic *β*-cell regeneration, insulin-releasing, and combating the problem of insulin resistance [8]. One of these plants is *Actinidia deliciosa* (AD) which pertains to the *Actinidiaceae* family. It is commonly known as kiwifruit, and the species is originated in China [9]. *A. deliciosa* is considered one of the extremely important sources of polyphenols, dietary fibers, protein, calcium, iron, and various vitamins especially vitamin C [10]. It has many biological activities including antioxidant, anticancer, amendment of platelet aggregation, regulation of lipids, lowering of blood pressure, and inflammation progress [10, 11]. Therefore, the present work objective is to investigate the possible antidiabetic, antihyperlipidemic, and antioxidant effects of *A. deliciosa* aqueous extract in STZ-induced diabetic rats.

## 2. Materials and Methods

*Actinidia deliciosa* (kiwifruit) was purchased from the local market, Alexandria, Egypt, and identified and authenticated by the Botany Department, Faculty of Science, Alexandria University. Streptozotocin was obtained by Sigma Chemical Company, USA.

### 2.1. Preparation of *A. deliciosa* Aqueous Extract

One hundred-gram kiwifruit was ground in a mill into fine particles, mixed with 250 distilled water, stirred at 25°C for one day, and filtered on filter paper. The filtrates were frozen then lyophilized, and the obtained powder was kept at -80°C until used for different assays [12].

### 2.2. GC-MS Analysis of *A. deliciosa*

Chemical constituents of lyophilized *Actinidia deliciosa aqueous extract* were identified using a Thermo Scientific GC/MS version (5) 2009 system with TG-5MS column (30 m×0.32 mm ID). The components of the ADAE were identified by mass fragmentation patterns, which were compared with the “National Institute of Standards and Technology (NIST)” mass spectral database (version 2), and their relative percentages were measured based on GC peak areas.

### 2.3. In Vitro Determination of Scavenging Antioxidant Properties of *A. deliciosa*

The DPPH free radical scavenging activity and total phenolic contents (TPC) of ADAE were determined using the method previously described by Gülçin [13] and Singleton et al. [14].

### 2.4. Diabetes Induction

Rats were starved during the night and provided with water freely. Diabetes was induced in male rats by an intraperitoneal single dose of 50 mg/kg body weight of streptozotocin freshly dissolved in 0.01 M citrate buffer (pH 4.5) [15]. The animals were allowed to drink glucose solution (5% *w*/*v*) overnight to avoid hypoglycemia which might be induced by streptozotocin; then, the animals were fed with a normal diet till the end of the study. A week later, a characteristic sign of diabetes was confirmed by the existence of a high glucose level of more than 250 mg/dl [16].

### 2.5. Animals and Experimental Protocol

Twenty-four male albino Wistar rats weighing 160-170 g were procured from the animal house of the Institute of Graduate Studies and Research, Alexandria University. Animals were handled following the guidelines of the National Institutes of Health (NIH) for laboratory animal welfare, and the experimental protocol was approved by the Local Ethics Committee and Animals Research (AU14-190323-2-7). The rats were housed in cages and maintained at a temperature of 22 ± 2°C, relative humidity of 40-60%, with a 12 h/12 h light/dark cycle and open access to pellet diet and water *ad libitum*. After a couple of weeks of adaptation, rats were randomly assigned to four groups with six animals each as follows: group I “control normal group” rats did not receive any drugs, group II received *A. deliciosa* aqueous extract (ADAE; 1 g/kg orally for 30 days) [17], rats of group III were treated with streptozotocin-induced DM (STZ; 50 mg/kg, i.p, single dose) [15], while group IV, diabetic rats received ADAE daily for 30 days after diabetes induction, respectively. Towards the end of the experimental time, rats were anesthetized using isoflurane then euthanized, and the blood and livers were taken for further analysis.

### 2.6. Blood and Tissue Samples

Blood specimens were singly gathered from rat aorta in nonheparinized glass tubes and left for 15 min. at 25°C to clot before being centrifuged at 3000 × g for 15 min. Sera were retained at -80°C until used. Liver tissues were immediately removed, weighed, and washed using saline solution. Tissues were homogenized in 0.01 M sodium phosphate buffer (pH 7.4) containing 1.15% KCl using a glass-Teflon Potter-Elvehjem homogenizer followed by centrifugation at 10,000 × g for 20 minutes at 4°C. The resultant supernatants were ready to use for different assays.

### 2.7. Determination of Oxidative Stress Markers

Thiobarbituric acid-reactive substances (TBARS), hydrogen peroxide (H_2_O_2_), reduced glutathione (GSH), superoxide dismutase (SOD), catalase (CAT), glutathione *S*-transferase (GST), glutathione peroxidase (GPx), and glutathione reductase (GR) were assessed in rat liver using kits from Biodiagnostic, Egypt.

### 2.8. Assessment of Liver Function Biomarkers and Lipid Profile

Alanine aminotransferase (ALT), aspartate aminotransferase (AST), lactate dehydrogenase (LDH), alkaline phosphatase (ALP) activities, protein content, and total bilirubin were assayed. Glucose level and lipid profile inclusive of “total cholesterol (TC), triglycerides (TG), high-density Lipoprotein-Cholesterol (HDL-C), low-density Lipoprotein-Cholesterol (LDL-C), and very-low-density lipoprotein-cholesterol (VLDL-C)” were estimated using Biodiagnostic Kits, Egypt. Serum insulin level was analyzed using an ultrasensitive rat insulin ELISA kit (Mercodia AB, Uppsala, Sweden) and a multiplate ELISA reader (Biorad-680, Bio-Rad Ltd., Japan).

### 2.9. Molecular Analysis by Real-Time PCR

Real-time qPCR was used to detect the relative expressions of peroxisome proliferator-activated receptor-gene (PPAR-*γ*), inflammation-related genes (interleukin 1*β* (IL-1*β*), tumor necrosis factor-*α* (TNF-*α*), nuclear factor kappa B (NF-*κ*B), and transforming growth factor-*β* (TGF-*β*)), proapoptotic genes (Bax and caspase3), and tissue inhibitor of metalloproteinase-1 (TIMP-1). Total RNA was isolated using a commercial kit (Gene JET RNA Purification Kit) following the manufacturer's protocol (Thermo Scientific, # K0731). Following the determination of RNA concentration by Nanodrop (Quawell, Q3000), cDNA was synthesized by reverse transcription using a commercial kit (RevertAid H Minus Reverse Transcriptase) as described in the manufacturer's instruction (Thermo Scientific, # EP0451). The qPCR was done using StepOnePlus Real-Time PCR System (Applied Biosystem) and a mixture of cDNA, 2x Maxima SYBR Green Master Mix (Thermo Scientific, # K0221), and gene-specific primers (Table 1). The results were normalized to glyceraldehyde-3-phosphate dehydrogenase (GAPDH) mRNA expression. Each sample was done three times along with nontemplate control in each plate. The thermal cycling and melting curve conditions were done as previously described. The relative gene expression was calculated using 2^–*ΔΔ*^Ct method.

### 2.10. Histopathological Examinations

Liver tissues were fixed in formalin solution (10%) for 2 days followed by dehydration in different concentrations of alcohol then embedded in paraffin. Paraffin sections were stained with hematoxylin and eosin; then, slides were pictured with a light microscope (Olympus BX 41, Japan).

### 2.11. Statistical Analysis

Data from different groups were represented as means ± standard errors (SEM) then analyzed utilizing the SPSS software (version 22, IBM Co., Armonk, NY). Comparison between groups was done through one-way ANOVA followed by Tukey's post hoc test. Significance at *P* value is ≤0.05.

## 3. Results

### 3.1. GC-MS Analysis of *A. deliciosa* Aqueous Extract

**Figure 1:**
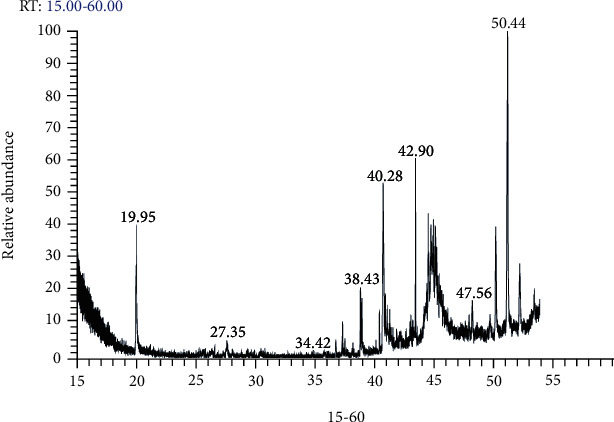
GC-MS chromatogram of *A. deliciosa* fruit aqueous extract.

The GC-MS analysis of *A. deliciosa* aqueous extract (Figure 1 and Table 2) revealed the appearance of different bioactive components (phytochemical constituents) that could collaborate with its antioxidant and therapeutic benefits including ascorbic acid, ᾅ-terpineol, oleic acid, vitamin E, hexanal, phytol, ethyl butanoate, and *α*-pinene. The labeling of the phytochemical constituents was approved based on the peak area, retention time (RT), and molecular formula.

### 3.2. In Vitro Antioxidant Capacity

In this study, ADAE effectively inhibited DPPH free radical scavenging activity with an IC_50_ value of 2.3 versus Vit C with an IC_50_ value of 3.5 mg/ml. The DPPH radical scavenging percentages of different concentrations (0.25–10 mg/ml) of ADAE extract were 30.1, 37.7, 49.7, 59.2, 63.8, 76.3, and 88.4%, respectively. Moreover, the total phenolic content was found equal to 12.8 ± 0.08 *μ*g GAE/mg DW.

### 3.3. Body and Liver Weights, Glucose, and Insulin Levels

In diabetic rats, blood glucose concentration was markedly increased, while insulin hormone level and body and liver weights, as well as body weight gain, were significantly decreased as compared to control. However, diabetic rats treated with ADAE showed significant alleviation to the changes detected in these parameters in comparison to the diabetic group. Rats that received ADAE only did not show any significant alteration in the mentioned parameters (Table 3).

### 3.4. Oxidative Stress Markers

The present results revealed a significant (*P* < 0.05) increase in TBARS (+44.55%) and H_2_O_2_ (+42.87%) levels and a significant reduction in GSH content (-45.53%) and SOD (-48.38%), CAT (-45.87%), GPx (-39.75%), GR (-44.82%), and GST (-45.30%) activities in diabetic rats' liver homogenates versus control, while diabetic rats supplemented with ADAE showed a significant restoration in these parameters as compared to diabetic ones. Supplementation with ADAE alone reduced the levels of TBARS (-23.96%) and H_2_O_2_ (-21.13%) and improved GSH content (+20.07%) as well as the antioxidant enzyme activities of SOD, CAT, GPx, GR, and GST by +20.69%, +19.58%, +17.72, +18.14, and +16.22% significantly and, respectively, as compared to the control group (Table 4).

### 3.5. Liver Function Biomarkers and Serum Lipid Profile

Data showed that liver LDH (+39.07%) activity and serum bilirubin level (+31.8%) were increased significantly (*P* < 0.05) while liver AST (-35.18%), ALT (-32.61%), and ALP (-37.54%) activities and protein content (-35.41%) decreased in STZ-treated rats as compared to control. Moreover, a significant restoration in enzyme activities, bilirubin level, and protein content in diabetic rats received ADAE versus diabetic group. ADAE supplementation alone did not affect any of these measured indices except LDH. Also, a significant (*P* < 0.05) elevation in TC, TG, LDL-C, and VLDL-C levels by +38.82, +35.85, +38.99, and+35.85%, respectively, was observed in diabetic rats, while HDL-C level was decreased by -37.56%. On one hand, in diabetic rats that received ADAE, a significant alleviation in TC, TG, LDL-C, HDL-C, and VLDL-C levels was observed when compared to the diabetic group. Supplementation of ADAE alone amended most of the measured parameters (Table 5).

### 3.6. ADAE Modulates mRNA Expression of PPAR-*γ* and TIMP1 in Hepatic Tissue

**Figure 2:**
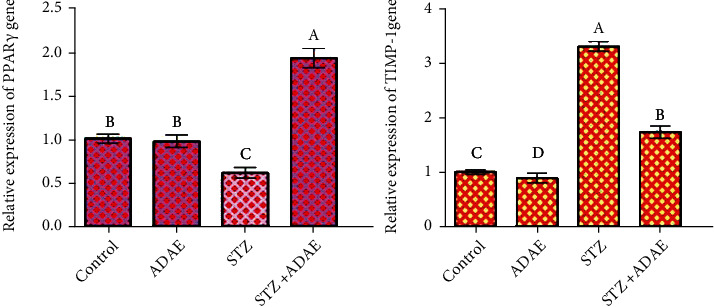
Effect of STZ and ADAE on the expression of liver PPAR-*γ* and antimigratory-related gene (TIMP-1 gene) by qRT-PCR. Data were normalized to the housekeeping gene (GAPDH) and are expressed as the mean fold change ± SEM. Samples were run in triplicate in three independent experiments. Groups with different letters are significantly different at *P* < 0.05.

The obtained RT-PCR results revealed a significant decrease in mRNA levels of peroxisome proliferator-activated receptor-gene (PPAR-*γ*) in liver tissue of STZ-treated rats compared to the control and ADAE groups. Diabetic rats given ADAE showed significant upregulation in this gene. The ADAE group exhibited insignificantly expression than the control group. Also, the antimigratory effect on a molecular level, qPCR was used to check changes in the relative expression of TIMP1. The obtained results revealed significant downregulation of TIMP1 in cells treated with ADAE relative to control cells (Figure 2).

### 3.7. Inflammation

**Figure 3:**
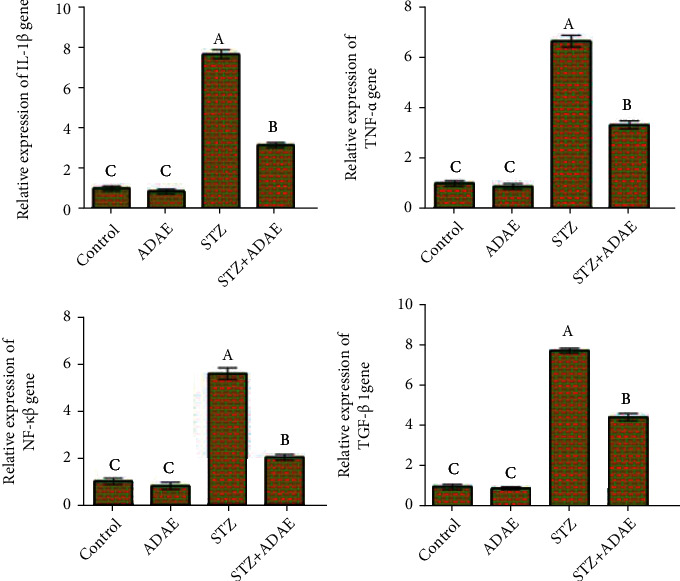
Effect of STZ and ADAE on the expression of inflammatory-related genes (IL-1*β*, TNF-*α*, NF-*κ*B, and TGF-*β*1) in the liver. Data are expressed as mean ± SEM. Samples were run in triplicate in three independent experiments. Groups with different letters are significantly different at *P* < 0.05.

The obtained qPCR results revealed significant increases in mRNA levels of proinflammatory cytokines IL-1*β*, TNF-*α*, NF-*κ*B, and TGF-*β*1 in liver tissue of STZ-treated rats compared to the control and ADAE groups. Diabetic rats administered ADAE led to significant downregulation in all genes, relative to the STZ group. The ADAE group exhibited a significantly lower expression than other groups (Figure 3).

### 3.8. Apoptosis

**Figure 4:**
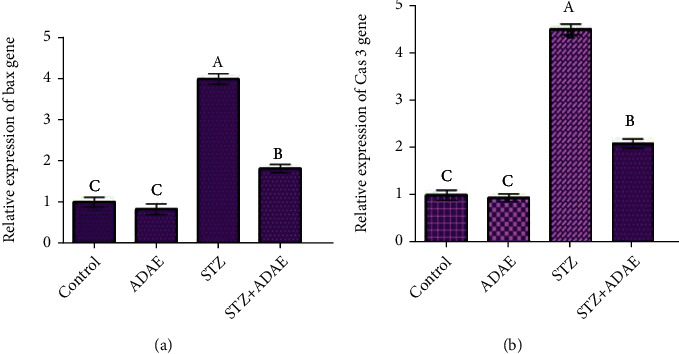
Effect of STZ and ADAE on the expression of liver apoptosis-related genes ((a) Bax; (b) caspase3 (Cas3)) by qRT-PCR. Data were normalized to the housekeeping gene (GAPDH) and are expressed as the mean fold change ± SEM. Samples were run in triplicate in three independent experiments. Groups with different letters are significantly different at *P* < 0.05.

DNA damage induced by STZ could suggest apoptosis in liver tissues. To check this possibility, qPCR was used to detect the apoptotic genes, Bax, and caspase3 expression. As expected, there was a significant upregulation of Bax and caspase3 in the liver tissue of rats treated by STZ relative to the control group (Figure 4). Treatment with ADAE led to a significant downregulation of those genes.

### 3.9. Liver Histopathology

**Figure 5:**
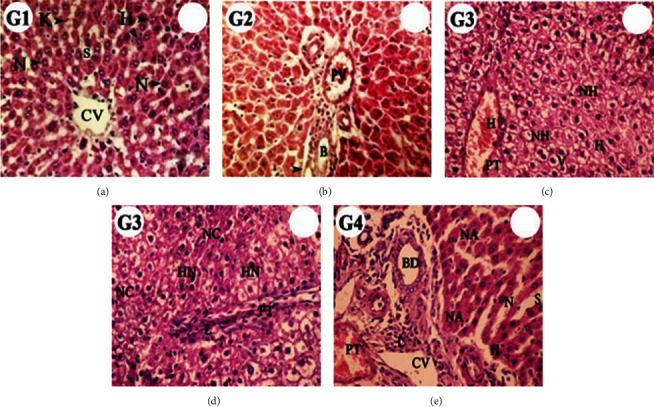
Photomicrographs of liver sections in different groups. Control (G1, (a)) and ADAE (G2, (b)) rats revealed the normal histological structures of hepatocytes (H) with nuclei (N), central vein (CV), sinusoids (S), Kupffer cells (K), branches of the hepatic portal vein (PV) and hepatic artery (arrow), and bile duct (B). Diabetic rats (G3, (c, d)) showed marked dilation and hemorrhage (H). The proliferating hepatocytes appear as normal architecture with round nuclei with vacuolated (V) and homogenous cytoplasm and few necrotic hepatocytes (NH). Also, crowded hepatocytes with hyperchromatic nuclei (HN) with eosinophilic cells as well as some area necrotic hepatocytes (NC), mild dilation of the portal tract (PT) with infiltrating lymphocytes (L), and migrated macrophage cells were observed. Diabetic rats supplemented with ADAE (G4, (e)) showed regenerative hepatocytes with rounded nuclei and few necrotic hepatocytes (NA), marked dilation of the sinusoid (S), and proliferating epithelial cells of the bile duct (BD) (H&E stain, 40x).

Light microscopic evaluation of the rat liver tissue sections of control (G1) and ADAE (G2) revealed the normal histological architecture of hepatocytes “with nuclei, central vein, sinusoids, and Kupffer cells.” In contrast, diabetic rat liver showed marked dilation and hemorrhage as well as hyperchromatic nuclei, necrotic hepatocytes with infiltrating lymphocytes, and migrated macrophage cells. The liver in diabetic rats administrated ADAE showed regenerative hepatocytes with little necrosis (Figure 5 and Table 6).

## 4. Discussion

The efficacy of *A. deliciosa* as a natural alternative and supplemental medicine for treating diabetes has gotten little attention. Therefore, the antioxidant, hypoglycemic, and hypolipidemic effectiveness, as well as anti-inflammatory and antiapoptotic role of ADAE supplementation in diabetic rats, were investigated in this study. The emergence of distinct bioactive components with high total phenolic contents and strong DPPH scavenging activity was discovered by GC-MS analysis of *A. deliciosa* aqueous extract, which could contribute to its antioxidant and therapeutic advantages [18]. Furthermore, phenolic compounds' antioxidant capabilities are due to their redox characteristics, as the phenol moiety assists them in acting as reducing agents, hydrogen donors, and singlet oxygen quenchers [19].

In diabetic rats, the marked changes in blood glucose concentration, insulin hormone level, and body and liver weights are related to the destruction of pancreatic *β*-cells which is accompanied by hyperglycemia, hyperlipidemia, and weight loss [20]. Furthermore, the fall in insulin levels in STZ-treated rats subsequently leads to a decrease in glucokinase activity, an insulin-sensitive enzyme that is deactivated in the diabetic rat liver. Also, insulin is fundamental in regulating glucose level homeostasis and promoting glycogenesis and glucose consumption. Moreover, excessive protein and lipid breakdown and/or dehydration may be linked to the body weight loss seen in diabetic rats [21]. Moreover, streptozotocin is a diabetogenic substance that causes diabetes in rats. STZ is taken up by pancreatic *β*-cell [22] via the glucose transporter, causing DNA fragmentation and cell death, as well as the generation of free radicals such as hydrogen peroxide, which leads to a decrease in insulin production and hyperglycemia.

In diabetic rats, the current study showed a significant increase in TBARS and H_2_O_2_ levels, as well as a significant reduction in GSH content and SOD, CAT, GPx, GR, and GST activities. These results support the hypothesis that hyperglycemia in diabetes is linked with oxidative stress, the formation of ROS, or the shortage of antioxidant defensive systems [23]. Furthermore, the considerable reduction in enzymatic and nonenzymatic antioxidants in diabetic rats could be related to an increase in ROS production, as well as the presence of H_2_O_2_ and other hydrogen radicals [4, 21, 24, 25]. Because glutathione is an important nonenzymatic antioxidant involved in maintaining cellular integrity, its depletion signals the advancement of cellular damage caused by free radicals. Superoxide dismutase catalyzes the conversion of superoxide anion to O_2_ and H_2_O_2_, which is then reduced to H2O by catalase as part of aerobic organisms' defense system against oxidative injury. The increased production of ROS by STZ could be the source of the decrease in both SOD and CAT activity. GPx protects membrane lipids from oxidative damage by catalyzing the reaction of hydroperoxides with GSH to create oxidized glutathione (GSSG). GST is important in the detoxification of xenobiotics to nontoxic metabolites [26]. Moreover, the inhibition in GR activity in STZ-treated rats was to compensate for the decreased GSH content by reducing GSSG. So, antioxidant enzymes which forbid the chain reaction of free radicals are crucial in recovering glucose levels in diabetic rats.

A significant alteration in AST and ALT is reported to be significant indicators of liver injury. This might be due to alterations in the cellular membrane's porosity as well as increased synthesis or decreased catabolism of aminotransferases. Through the Krebs cycle, these modifications also boost ketogenesis and gluconeogenesis, which are seen in diabetes. Moreover, the alterations in LDH and ALP activities showed that diabetes may induce hepatic dysfunction via liver necrosis [27], which results in the release of these enzymes into the bloodstream. Also, the elevation in serum total bilirubin may arise from reduced liver uptake, conjugation, or increased bilirubin generation from hemolysis. Protein is vulnerable to free radical damage, and its reduction could be caused by a change in protein metabolism [24]. Furthermore, diabetic rats showed vascular congestion, mononuclear cellular infiltration, and distorted cellular structure, indicating liver injury [21, 28].

Changes in lipid profile were also observed in STZ-treated rats [4, 29]. Hypercholesterolemia is caused by a decrease in cholesterol absorption, an increase in cholesterol manufacturing in the liver, a lack of membrane integrity, and a blockage of the hepatic bile ducts, resulting in reduced or no bile secretion to the duodenum. An increased level of TGs could also be attributable to an increase in the flux of free fatty acids and a disruption in the mobilization of VLDL-C from circulation. In diabetes, lipoprotein lipase activity is inhibited due to insulin insufficiency, which promotes hypertriglyceridemia. Furthermore, elevated LDL-C is linked to saturated fat, which reduces the number of LDL receptors, resulting in the liver's inability to catabolize LDL-C [30]. HDL-C also plays an important function in cholesterol leakage from tissues, transporting it back to the liver for bile acid clearance.

Inflammation plays an important role in host defense by generating proinflammatory cytokines that protect the host from harm, including TNF-*α* and IL-1*β*. Tissue damage, fibrosis, and loss of cellular function are all symptoms of chronic inflammatory diseases. Growing evidence links a low-grade chronic inflammatory state to DM complications, particularly liver-related problems [31]. These conditions are further exacerbated by the combined action of a complex regulatory network of cells and mediators designed to resolve inflammatory responses, mainly macrophages. Insulin resistance will be aggravated as a result of the rise in circulating cytokines [32–34]. Thus, inflammation may be a major causal factor rather than a risk factor for noncommunicable diseases like diabetes. For example, IL-1*β* together with TNF-*α* is responsible for the development of DM types 1 and 2 through inhibition of glucose-induced insulin secretion and impairment of *β*-cell function, thereby decreasing the biosynthesis of insulin and inducing apoptotic cell death [34]. In addition, IL-1*β* may also increase T- and *β*-cell proliferation and cause the release of adhesion molecules, as well as prompt the production of other cytokines and proinflammatory mediators, all of which can be harmful to the liver [35–37]. The activation of the c-Jun N-terminal kinase (JNK) pathway and nuclear translocation of NF-*κ*B are the most common biological responses to IL-1 [36]. As a result, activation of NF-*κ*B in DM may trigger a cascade of negative events by increasing proinflammatory cytokines [36, 38, 39]. Furthermore, NF-*κ*B may enhance the generation of ROS and RNS, as well as increase LPO, resulting in increased liver tissue damage [32]. Tissue inhibitor of metalloproteinase-1 (TIMP-1) is a glycoprotein produced by macrophages and connective tissue cells that inhibits the action of matrix metalloproteinases (MMPs), particularly MMP-16, and plays a key role in the fibrous liver's collagen degradation [40]. Hyperglycemia causes an increase in TGF-*β*1 and TIMP-1 secretion. In type 2 DM and hepatogenous DM, insulin resistance is common, and the insulin level increases, boosting the formation of oxygen free radicals and stimulating the release of inflammatory mediators including tumor necrosis factor-*α* (TNF-*α*) [41].

Improved insulin sensitivity can be obtained through systemic insulin sensitization or direct action of the peroxisome proliferator-activated receptor (PPAR-*γ*) on the transcription of genes involved in glucose elimination that provide evidence for PPAR-*γ*'s direct influence on glucose metabolism. Furthermore, PPAR-*γ* may have a direct effect on the liver and pancreatic *β*-cells to improve glucose homeostasis [42]. Endothelial function is improved by PPAR-*γ* ligands boosting endothelial NO bioavailability, which is achieved in part by changing endothelial superoxide metabolism via NOX inhibition and SOD1 activation [43]. Furthermore, it has been demonstrated that the PPAR-*γ* ligand can reduce NF-*κ*B/p65 activation and NOX4 expression in high-glucose-induced oxidative stress [44]. As a result, PPAR-*γ* may operate as an additional regulator of redox signaling in the cardiovascular system, in addition to its role in limiting lipid accumulation [42]. There is strong evidence that free radicals play a key role in DNA damage and cytotoxicity induced by STZ [45].

Dietary antioxidants including vitamins C and E, as well as polyphenols, may help to protect DNA by scavenging reactive species or by enhancing endogenous antioxidant activity. Endogenous oxidation of DNA pyrimidines and purines was linked to kiwifruit consumption, showing that a small amount of kiwifruit, as found in a typical diet, may protect against DNA damage that would otherwise result in mutations [46]. Oral administration of ADAE decreased the glucose level with a concomitant increase in insulin due to improvement in *β*-cell number [5]. ADAE also reduced lipid peroxidation in diabetic rats, indicating that it can protect against the damaging effects of ROS by either limiting their production or altering their aggressiveness. Moreover, ADAE restored the changes in antioxidant enzyme activities which could be linked to its role in the inhibition of ROS production. In addition, increased GSH content aids in the elimination of ROS, the preservation of cell integrity, and the protection of cellular constituents versus oxidation. Kiwifruit is also a good source of antioxidants and natural pancreatic lipase and *α*-glucosidase inhibitors [47]. Similarly, diabetic rats given onion as part of their diet had considerably higher body weights and antioxidant enzyme activity [25]. In diabetic rats, hesperetin, a citrus flavonoid, also attenuates high blood glucose and lipid levels through boosting antioxidant proficiency. Moreover, it promotes the revitalization of islet cells and further provokes insulin discharge by protecting *β*-cells from free radicals' utilization by preventing the glycation of antioxidant enzymes [21]. Also, phlorotannins isolated from brown seaweed declined glucose level and lipid peroxidation and improved antioxidant capacity, insulin, and hepatic GSH [48]. Furthermore, flavonoids are extensively proved to produce a valuable protective effect through enforcement of the antioxidant status [49]. Furthermore, *A. deliciosa* was found to be effective in repairing the distorted cellular organization seen in diabetic rats' hepatic tissue architecture [28]. In addition, kiwifruit has a low glycemic index, making it good for diabetics. Also, fiber-rich foods, like kiwifruit, are good for keeping the blood sugar levels of diabetic patients under control. Moreover, it contains several components that have potential anti-inflammatory properties in vivo, as evidenced by finding in the stomach and duodenum of rats fed with kiwifruit at concentrations that decrease proinflammatory genes [50]. So, this study is a prosperous trial for the use of *A. deliciosa* as a complementary remedy and exquisite antioxidant in diabetes disease.

## 5. Conclusion

In conclusion, this study reveals that ADAE significantly modulates hyperglycemia, hyperlipidemia, and oxidative stress in diabetic rats. Furthermore, ADAE supplementation improved liver function biomarkers, antioxidant status, and liver architecture by inhibiting ROS generation. In diabetic rats' hepatic tissue, it also upregulates PPAR-*γ* while downregulating apoptotic and inflammatory gene expression. As a result, *A. deliciosa* has a strong antioxidant impact and could be employed as a supplemental therapy in the treatment of diabetes mellitus.

## Figures and Tables

**Table 1:** Forward and reverse primer sequence used in RT-PCR.

Gene	Forward primer	Reverse primer
Inflammatory-related genes		
IL-1*β*	CACCTCTCAAGCAGAGCACAG	GGGTTCCATGGTGAAGTCAAC
TGF-*β*1	AAGAAGTCACCCGCGTGCTA	TGTGTGATGTCTTTGGTTTTGTCA
TNF-*α*	GCATGATCCGCGACGTGGAA	AGATCCATGCCGTTGGCCAG
NF-*κ*B	CCTAGCTTTCTCTGAACTGCAAA	GGGTCAGAGGCCAATAGAGA
Glycolated-related gene		
TIMP-1	GGGCTTCACCAAGACCTACA	TGCAGGGGATGGATAAACAG
PPAR-*γ*	CACGAAGAGCCTTCCAACTC	TATGAGACATCCCCACAGCA
Apoptotic-related genes		
Bax	GGACGAACTGGACAGTAACATGG	GCAAAGTAGAAAAGGGCGACAAC
Caspase3	GAAGCGAATCAATGGACTCTGG	GACCGAGATGTCATTCCAGTGC
Housekeeping		
GAPDH	GGTGAAGGTCGGAGTCAACG	TGAAGGGGTCATTGATGGCAAC

**Table 2:** Major components found in *A. deliciosa* extract according to the retention time.

No.	Compound name	Molecular formula	Molecular weight	RT	KI
1	Methyl ethanoate	C_3_H_6_O_2_	74	19.95	832
2	Methyl butanoate	C_5_H_10_O_2_	102	27.35	987
3	*α*-Pinene	C_10_H_16_	136	34.42	982
4	Toluene	C_7_H_8_	92	37.72	1036
5	Ethyl butanoate	C_6_H_12_O_2_	116	38.43	1040
6	Phytol	C_20_H_40_O	256	39.96	930
7	Hexanal	C_6_H_12_O	100	40.28	1081
8	Vitamin E	C_29_H_50_O_2_	430	42.90	1040
9	Oleic acid	C_6_H_12_O_6_	282	47.56	1179
10	ᾅ-Terpineol	C_10_H_18_O	154	49.23	1198
11	Ascorbic acid	C_38_H_68_O_8_	652	50.44	1270

RT: retention time; KI: retention index: Kovats retention index.

**Table 3:** Body and liver weights, blood glucose, and insulin levels in different groups.

Parameters	Control	ADAE	STZ	STZ + ADAE
Glucose (mg/dl)	108 ± 2.70^c^	101 ± 3.12^c^	356 ± 9.82^a^	195 ± 5.60^b^
Insulin hormone (*μ*U/ml)	11.43 ± 0.352^a^	10.93 ± 0.281^a^	6.52 ± 0.217^c^	9.38 ± 0.170^b^
Initial body weight (g)	155 ± 2.69	159 ± 3.02	156 ± 2.61	155 ± 2.16
Final body weight (g)	217 ± 2.24^a^	224 ± 4.67^a^	174 ± 3.82^c^	198 ± 3.82^b^
Weight gain (g)	62 ± 4.21^a^	65 ± 5.96^a^	18 ± 3.97^c^	42 ± 2.78^b^
Liver weight (g)	6.65 ± 0.254^a^	6.91 ± 0.275^a^	4.78 ± 0.126^c^	5.76 ± 0.109^b^
Liver/body weight ratio	3.06 ± 0.089^a^	3.10 ± 0.190^a^	2.76 ± 0.117^b^	2.92 ± 0.096^a^

Values are expressed as means ± SE (*n* = 6). Values within a row not sharing a common superscript letter were significantly different. Statistically significant variations are compared: ADAE and STZ groups compared vs. control while STZ + ADAE group compared vs. STZ group. Weight gain = Final body weight–Initial body weight. Liver − body weight ratio = (Liver weight/body weight × 100).

**Table 4:** Oxidative stress markers in different groups.

Parameters	Control	ADAE	STZ	STZ + ADAE
TBARS (nmol/g tissue)	29.64 ± 0.99^c^	22.54 ± 0.842^d^	42.85 ± 1.527^a^	35.46 ± 1.437^b^
H_2_O_2_ (*μ*mol/g tissue)	68.52 ± 2.5^c^	54.04 ± 2.15^d^	97.89 ± 287^a^	86.35 ± 2.57^b^
GSH (mmol/mg protein)	2.17 ± 0.07^b^	2.60 ± 0.11^a^	1.18 ± 0.04^d^	1.56 ± 0.05^c^
SOD (U/mg protein)	76.55 ± 2.404^b^	92.39 ± 2.29^a^	39.51 ± 1.497^d^	60.66 ± 1.891^c^
CAT (*μ*mol/h/mg protein)	42.73 ± 1.62^b^	51.10 ± 1.22^a^	23.13 ± 0.82^d^	32.25 ± 0.87^c^
GST (*μ*mol/hr/mg protein)	1.10 ± 0.039^b^	1.28 ± 0.0508^a^	0.6013 ± 0.0219^d^	0.79 ± 0.0302^c^
GPx (U/mg protein)	0.93 ± 0.03^b^	1.09 ± 0.037^a^	0.56 ± 0.02^d^	0.74 ± 0.028^c^
GR (U/mg protein)	1.22 ± 0.044^b^	1.44 ± 0.049^a^	0.67 ± 0.023^d^	0.89 ± 0.031^c^

Values are expressed as means ± SE (*n* = 6). Values within a row not sharing a common superscript letter were significantly different. Statistically significant variations are compared: ADAE and STZ groups compared vs. control while STZ + ADAE group compared vs. STZ group.

**Table 5:** Liver function biomarkers and lipid profile in different groups.

Parameters	Control	ADAE	STZ	STZ + ADAE
AST (U/mg protein)	90 ± 1.08^a^	95 ± 3.02^a^	59 ± 1.84^c^	72 ± 2.53^b^
ALT (U/mg protein)	124 ± 4.93^a^	134 ± 4.64^a^	84 ± 3.11^c^	100 ± 3.07^b^
ALP (U/mg protein)	325 ± 11.41^a^	311 ± 11.70^a^	203 ± 7.16^c^	258 ± 9.97^b^
LDH (U/mg protein)	811 ± 25.91^c^	713 ± 25.60^d^	1128 ± 31.72^a^	950 ± 34.99^b^
Protein (mg/g tissue)	193 ± 7.30^a^	203 ± 8.55^a^	125 ± 4.58^c^	150 ± 4.99^b^
Total bilirubin (mg/dl)	0.763 ± 0.032^c^	0.740 ± 0.022^c^	1.01 ± 0.034^a^	0.885 ± 0.023^b^
TC (mg/dl)	144 ± 4.87^c^	120 ± 4.50^d^	200 ± 7.19^a^	171 ± 5.91^b^
LDL (mg/dl)	49.04 ± 1.98^c^	44.72 ± 1.59^c^	68.16 ± 1.75^a^	58.88 ± 1.97^b^
HDL (mg/dl)	42.87 ± 2.01^b^	47.50 ± 1.41^a^	26.77 ± 1.13^d^	34.30 ± 1.09^c^
TG (mg/dl)	107 ± 4.04^c^	88 ± 2.07^d^	146 ± 5.45^a^	129 ± 5.23^b^
VLDL (mg/dl)	21.47 ± 0.81^c^	17.53 ± 0.41^d^	29.17 ± 1.09^a^	25.76 ± 1.05^b^

Values are expressed as means ± SE (*n* = 6). Values within a row not sharing a common superscript letter were significantly different. Statistically significant variations are compared: ADAE and STZ groups compared vs. control while STZ + ADAE group compared vs. STZ group.

**Table 6:** Histological score in the liver.

	Control	ADAE	STZ	STZ + ADAE
Necrotic hepatocytes	-	-	+	+
Hyperchromatic nuclei	-	-	+++	+
Vacuolation (infiltration)	-	-	++	+
Hemorrhage	-	-	+	-

–: no lesion; +: mild; ++: moderate; +++: high.

## Data Availability

All the data used to support the findings of this study are included within the article.
